# Cyclodextrin Polymer-Loaded Micro-Ceramic Balls for Solid-Phase Extraction of Triazole Pesticides from Water

**DOI:** 10.3390/ijms25041959

**Published:** 2024-02-06

**Authors:** Xiaobo Yang, Lingli Yu, Shuqi Chen, Miaochang Liu, Qian Miao, Huayue Wu, Wenxia Gao

**Affiliations:** 1 College of Chemistry and Materials Engineering, Wenzhou University, Wenzhou 325027, Chinamiaoqian@wzu.edu.cn (Q.M.); huayuewu@wzu.edu.cn (H.W.); 2 College of Pharmacy, Chengdu University, Chengdu 610106, China

**Keywords:** β-CD polymer, adsorption, solid-phase extraction, triazole pesticides, recycling efficiency

## Abstract

A citric acid cross-linked β-cyclodextrin (β-CD) polymer was synthesized and loaded on micro-ceramic balls to fabricate the solid-phase adsorbents (P-MCB) for adsorption and extraction of triazole pesticides from water. The stability of β-CD polymer and P-MCB was investigated in solutions with different pH values at different temperatures. The adsorption properties and the influence of kinetics, sorbent amount, pesticide concentration, and temperature on the adsorption capacity were evaluated. The results showed P-MCB had favorable adsorption of 15.98 mg/g flutriafol in 3.5 h. The equilibrium data followed the Freundlich equation, and the adsorption of flutriafol and diniconazole followed the second-order kinetics. The recovery rate of P-MCB for triazole pesticides in water was satisfactory, and the recovery rate was still 80.1%, even at the 10th cycle. The P-MCB had good stability, with a degradation rate of 0.2% ± 0.08 within 10 days, which could ensure extraction and recycling.

## 1. Introduction

Triazole pesticides (flutriafol, diniconazole, penconazole, hexaconazole, tebuconazole, and difenoconazole) are widely used in the world as fungicides to control diseases of turf, vegetables, citrus, field crops, ornamental plants, and so on [1,2]. Though these triazole pesticides play an important role in agricultural production, more and more attention has been paid to the problem of the non-rational use of these pesticides, the potential threat to human health, and the pollution of the environment [3,4]. Triazole pesticides enter the human body through respiratory tract, skin contact, and food contaminated with them, causing endocrine toxicity in mammals [5]. Therefore, in numerous countries, residues of triazole pesticides in vegetables and fruit are strictly limited, and the maximum residue limit is 0.01 mg/kg. In addition, the adsorption of triazole pesticides in water is widely considered by researchers [6].

There are three strategies for removing toxic pollutants from wastewater: physical, chemical, and biological methods. The settling method is one of the important physical methods, which separates particles from the fluid through gravity settling. During the water treatment process, the decrease in water flow velocity causes suspended particles to remain stable under static conditions, and then the particles settle under the action of gravity [7]. Filtration is the process of removing contaminants according to their size, which is the first step in removing hazardous substances [8]. In terms of biology, microorganisms can decompose organic matter in wastewater through two different biological processes, namely biological oxidation and biosynthesis [9]. In order to treat toxic pollutants in water effectively, chemical methods are used to supplement the shortcomings of physical or biological methods [10]. Chemical adsorption is an important method due to the presence of functional groups on the surface of the adsorbent, which can form electrostatic interactions or chemical bonds with pollutants to adsorb them. Zhang et al. prepared the magnetic biochar (MMABC) for chemical adsorption to remove Cr (VI) from water, and the effect was significant [11]. Compared with other removal methods, adsorption has advantages in terms of cost-effectiveness and operation.

Due to the lipophilicity and stability of triazole pesticides, they are easy to accumulate and transport in soil and water. Therefore, there is an urgent need for an effective adsorbent to eliminate triazole pesticides. Liu et al. prepared a novel magnetic copper-based metal organic framework material (M-MOF-199) using Fe_3_O_4_ graphene oxide (GO) nano-composites [2]. Five triazole pesticides were solid-phase extracted from water samples, and the results showed that M-MOF-199 was a promising adsorbent for magnetic dispersion solid-phase extraction and removal of triazole pesticides from environmental water samples. Yi et al. [12] treated microcrystalline cellulose (MCC) with sulfuric acid to obtain magnetic partially carbonized cellulose nanocrystals (MPC-CNC) and then loaded magnetic Fe_3_O_4_ nanoparticles, which could be used for magnetic solid-phase extraction of triazine and triazole pesticides from water. The results show that MPC-CNC@Fe_3_O_4_ was a feasible adsorbent.

Cyclodextrin (CD) derivatives have recently gained more attention as an adsorbent for removing organic pollutants and heavy metal ions from water [13,14]. CDs are cyclic oligosaccharides produced by the digestion of starch by Bacillus amyloliquefaciens [15]. Three natural CDs (α-, β-, and γ-CD) have 6, 7, and 8 α-1,4-glycosidic bonds that form a cyclic structure with a hydrophilic outer shell and a hydrophobic inner cavity, capable of accommodating various organic guest molecules [16]. Pollutants or various organic molecules can be captured through host-guest interactions, forming inclusion complexes [17,18]. The formation of inclusion complexes between cyclodextrin and organic molecules depends on multiple factors. The host cavities of organic molecules and CDs are adjustable in size and shape. Due to their suitable adjustability and microenvironment, non-polar molecules are easily regulated to form inclusion complexes in the lipophilic cavities of CDs without forming or breaking covalent bonds. Alsbaiee et al. [19] researched a β-CD cross-linked with rigid aromatic groups; the mesoporous β-CD polymer had a high surface area for quickly isolating various organic micropollutants. This porous cyclodextrin is superior to activated carbon in removing mixtures of organic micropollutants with environmental-related concentrations. Chin et al. [20] reported the favorable thermodynamical adsorption of the aromatic ring of ARS into the secondary hydroxyl edge of the CD cavity. Raoov et al. [21] synthesized functional β-CD ionic liquid polymer (β-CD BIMOTs TDI); it enhanced adsorption capacity and had a high removal rate for phenol and As (V).

The general method for obtaining β-CD polymers is to cross-link β-CD molecules with bifunctional cross-linkers, such as epichlorohydrin, ethylene glycol bis(epoxypropyl)ether, and so on [22,23]. However, it is difficult to recycle these β-CD derivatives dispersed in the water phase after the adsorption of pollutants. A recyclable adsorption column was obtained by preparing a new β-CD polymer, which was loaded on porous ceramic balls to explore the solid-phase extraction of triazole pesticides in this work.

Though polymer adsorbent materials have been widely used in wastewater treatment, they still have some problems, such as poor adsorption selectivity, low adsorption capacity, secondary treatment, and secondary pollution [24,25]. It is especially difficult to quickly and effectively separate the polymer material from the wastewater and reuse it, which limits the application [26]. A common way to solve this problem is to fix the polymer material onto the carrier. Ceramic balls have a porous plate structure, as well as the characteristics of strong chemical stability, wear resistance, corrosion resistance, high temperature resistance, and low cost, which are used widely as the filter medium. With the growing demand for high-performance and high-stability adsorbent materials, ceramic balls, as adsorbents for various gases and liquids, have achieved good results in environmental protection, such as sewage treatment, waste gas treatment, and air purification. However, in terms of high selectivity, ceramic balls also need to be modified to achieve better performance [27,28,29]. Therefore, it is of great significance to load polymer materials on ceramic balls.

This paper investigated the influence of kinetics [30], sorbent amount [31], pesticide concentration, and temperature on the adsorption capacity of polymer-supported materials [32]. The equilibrium data has been analyzed using Freundlich isotherms [33], and the characteristics and parameters for each isotherm have been determined. The recycling efficiency of the β-CD polymer loaded micro-ceramic balls (P-MCB) for solid-phase adsorption and extraction of triazole pesticides from water was also evaluated.

## 2. Results and Discussion

### 2.1. Preparation and Characterization of Adsorbents

**Figure 1:**
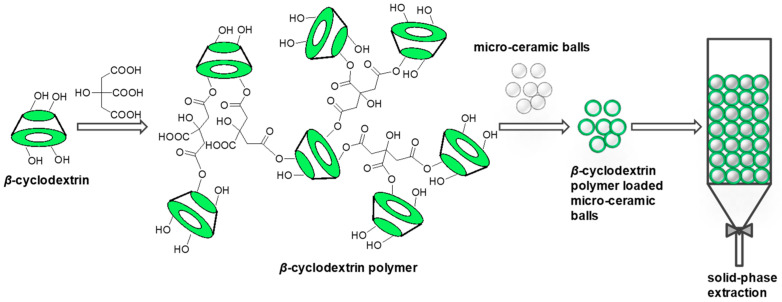
Preparation of β-CD polymer loaded micro-ceramic balls for solid-phase extraction.

**Figure 2:**
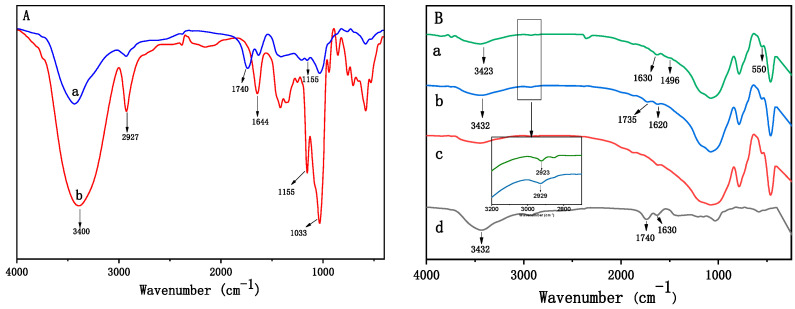
IR spectra (**A**) β-CD polymer (a) and β-CD (b); (**B**) β-CD polymer loaded micro-ceramic balls (a), mechanical mixing of β-CD polymer and micro-ceramic balls (b), native micro-ceramic balls (c), β-CD polymer (d).

The adsorbent was prepared as shown in Figure 1. β-CD was cross-linked by nontoxic citric acid, and the citric acid cross-linked β-CD was further loaded onto micro-ceramic balls to obtain the sorbent. The infrared spectra of β-CD and β-CD polymer are presented in Figure 2. β-CD showed peaks at 3400 cm^−1^ and 2927 cm^−1^ due to the O–H and C–H stretching vibrations. In addition, peaks at 1644 cm^−1^, 1155 cm^−1^, and 1033 cm^−1^ corresponded to C–O, C–O–C of glucose units and C–O–C of β-CD were observed (Figure 2A). In the spectrum of the β-CD polymer, a new peak appeared at 1740 cm^−1^, which belonged to the vibration of the –COO– group, proving that β-CD was grafted with citric acid (Figure 2A). The O–H stretching vibration of β-CD polymer loaded micro-ceramic balls corresponding to a hydroxyl group and a carboxyl group at 3423 cm^−1^ was obviously weakened, which might be due to the condensation of metal ions with –COOH. The characteristic peak at 550 cm^−1^ could be attributed to the tensile vibration of the metal–OH (M–OH) bond, which was absent in the FT-IR spectrum of the β-CD polymer. Moreover, the absorption peak of –OH was 3427 cm^−1^, the absorption peak of C-H tensile vibration was 2923 cm^−1^, the absorption peak of –COO was 1630 cm^−1^, and the absorption peak of C–O was 1496 cm^−1^, all of them were red-shifted. This might be due to the conjugation of the metal groups in the micro-ceramic spheres with the β-CD polymer, which weakened the chemical bond properties of the original group, thereby reducing the force constant and the absorption frequency (Figure 2B) [34,35,36].

**Figure 3:**
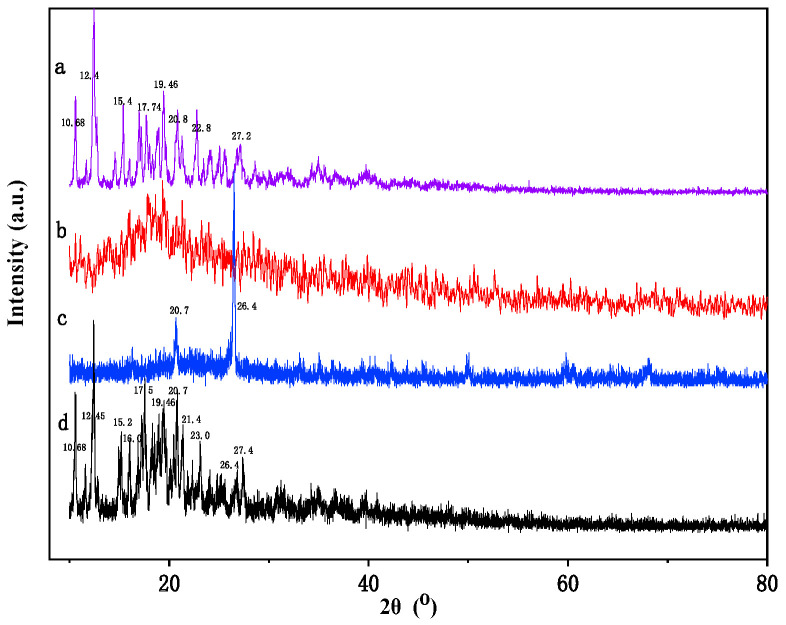
XRD spectra of β-CD (a), β-CD polymer (b), native micro-ceramic balls (c), β-CD polymer loaded micro-ceramic balls (d).

The XRD is presented in Figure 3. The β-CD monomer was crystalline, and its main diffraction peaks appeared at 10.68°, 12.4°, 15.4°, 17.74°, 19.46°, 20.8°, 22.8°, 27.2° (Figure 3a) [37,38]. In contrast, the XRD spectrum of β-CD polymer had undergone significant changes, with a decrease in diffraction intensity and a change in peak shape from a sharp peak to a diffuse peak, indicating that the crystal of β-CD was destroyed and transformed into semi-crystalline or amorphous after polymerization (Figure 3b) [39]. The main component of the original micro-ceramic ball was Al_2_O_3_, with its main diffraction peaks at 20.7° and 26.4° (Figure 3c) [40]. β-CD polymer loaded micro-ceramic balls exhibited significant diffraction peaks at 10.68°, 12.45°, 15.2°, 16.0°, 17.5°, 19.46°, 20.7°, 21.4°, 23.0°, 26.4°, and 27.4°, and with a peak envelope, indicating that the micro-ceramic balls adsorb the β-CD polymer (Figure 3d).

**Figure 4:**
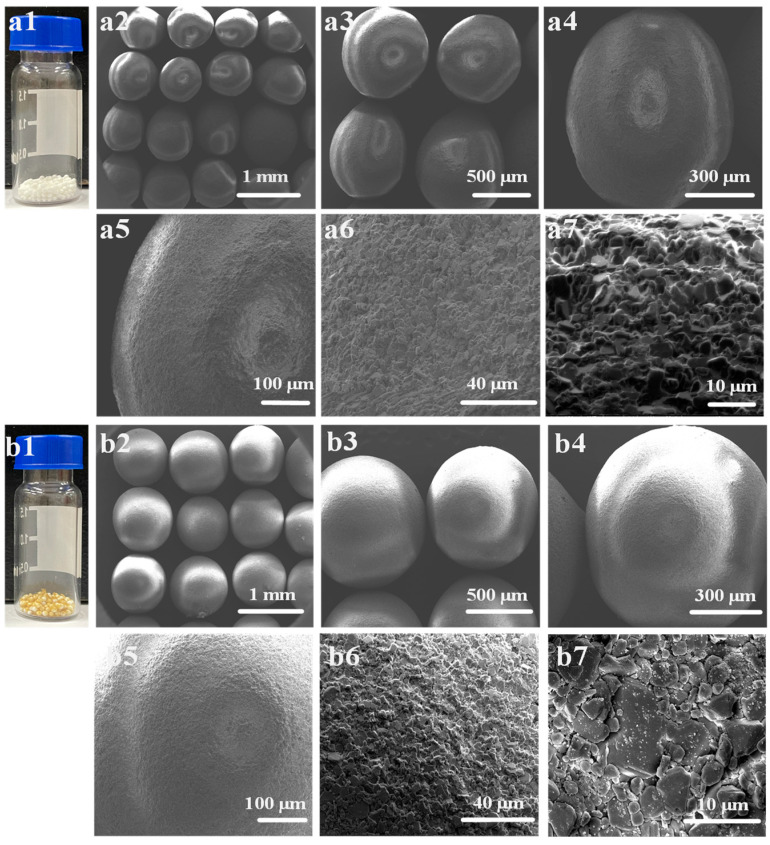
Photos and SEM images of native micro-ceramic balls and β-CD polymer loaded micro-ceramic balls. (**a1**) photo of native micro-ceramic balls, (**a2**–**a7**) SEM images of native micro-ceramic balls with different magnification, (**b1**) photo of β-CD polymer loaded micro-ceramic balls, (**b2**–**b7**) SEM images of β-CD polymer loaded micro-ceramic balls with different magnification.

The photos and SEM images of the original micro-ceramic spheres and the micro-ceramic spheres loaded with β-CD polymer are shown in Figure 4. The micro-ceramic ball was white in appearance (Figure 4(a1)). Their surfaces were relatively smooth, the shape of the balls was not very regular, and the particle size distribution was between 600–900 µm (Figure 4(a2–a4)). The surface of the primary micro-ceramic ball was layered (Figure 4(a5,a6)), and no nanoparticles were on the surface of the layered structure (Figure 4(a7)). When the natural micro-ceramic spheres were coated with β-CD polymer, the color of the micro-ceramic spheres changed to yellow (Figure 4(b1)). The shape of these micro-ceramic spheres loaded with β-CD polymer became more regular and full (Figure 4(b2–b4)), and the surface was also layered (Figure 4(b5,b6)), and the nanoparticles were found on the surface of the layered structure (Figure 4(b7)). Since the metal groups in the micro-ceramic spheres condensed with the –COOH in the β-CD polymer, the nanoparticles of the β-CD polymer could be adsorbed on the micro-ceramic spheres.

### 2.2. Degradation of β-CD Polymer

**Figure 5:**
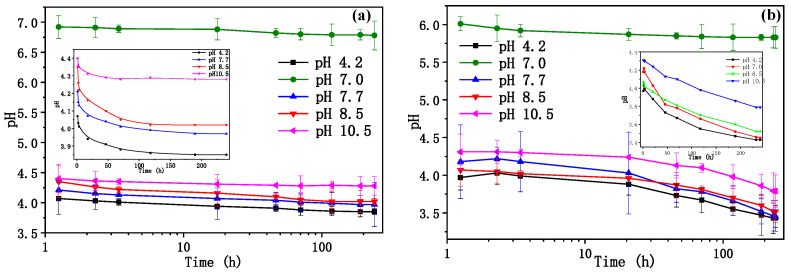
The pH values of β-CD polymer solutions as a function of time during degradation with different initial pH values (pH = 2.24, 4.22, 6.70, 7.41, 8.54, 10.50), at different temperatures of 25 °C (**a**) and 35 °C (**b**).

Degradation of β-CD polymer was carried out by adding polymer to the solution with different pH values (pH = 2.24, 4.22, 6.70, 7.41, 8.54, 10.50) at 2 5 °C and 35 °C (Figure 5). pH values of these solutions were measured at prescribed time intervals until there was no distinguish change, as shown in Figure 5. At 25 °C, the pH values of the solution containing polymers decreased with the extension of time, and there was a pH drop in the acidic and alkaline polymer solutions for the first 100 h (Figure 5a), and the pH change was not obvious at 200 h (Figure 5a inset). At 35 °C, the decreasing trend of pH with time was more obvious than that at 25 °C, and the degradation efficiency also increased with the decrease of pH (Figure 5b). The polymer was stable in a neutral environment at room temperature. Degradation was observed both in acidic and alkaline environments; there was 10.4% and 12.8% of polymer degraded in the solution with pH 4.2 and pH 10.5, respectively (Table 1). Moreover, higher temperature improved the degradation of the polymer, the degraded polymer was increased to 23.2% and 22.9% in the solution with pH 4.2 and pH 10.5, respectively. This polymer was more stable in a neutral medium at low temperatures. The polymer was further loaded onto the micro-ceramic balls, and the investigation of the β-CD polymer loaded micro-ceramic balls (P-MCB) showed that they were stable with few degradation of 0.2 ± 0.08% in 10 d in neutral medium at room temperature. This ensured the usage of P-MCB for solid-phase extraction.

### 2.3. Adsorption Isotherm Models

**Figure 6:**
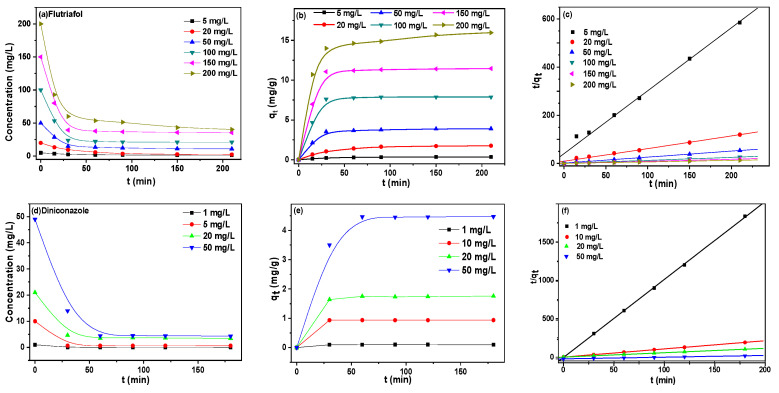
Concentration of flutriafol (**a**) and diniconazole (**d**) solution versus the contact time onto β-CD polymer, effect of contact time on the adsorption of flutriafol (**b**) and diniconazole (**e**), pseudo-second-order kinetics of flutriafol (**c**) and diniconazole (**f**). (conditions: initial flutriafol concentration = 200, 150, 100, 50, 20, 5 mg/L; initial diniconazole concentration = 50, 20, 5, 1 mg/L; volume, 10 mL; β-CD polymer mass = 100 mg; temperature: 25 °C).

Initial pesticide concentration in the solution was varied to investigate its effect on the adsorption capacity. Figure 6a shows the relationship between the equilibrium concentration (*C_e_* in mg/L) of six different initial concentrations of fluorotriol and the contact time. With the increase of contact time, the concentration of fluorotriol decreased and finally reached the equilibrium concentration. Most concentrations reached equilibrium after 200 min. For diconazole (Figure 6d), the same phenomenon was observed, and the equilibrium concentration of diconazole also decreased with increasing absorption time and reached the equilibrium concentration at 60 min. Studies were also conducted for various time intervals, to determine when adsorption equilibrium was reached and the maximum removal of pesticide was attained. The amount of pesticide adsorbed at equilibrium, *q_e_*, was calculated from the mass balance equation given by [41]:

*q_e_* = *V*(*C_o_* – *C_e_*)/*m*, (1)

where *C_o_* was the initial compound concentration in the liquid phase (mg/L); *C_e_* was the liquid phase compound concentration at equilibrium (mg/L); *V* was the volume of compound solution used (L); and *m* was the mass of sorbent used (g).

Figure 6b shows the amount of flutriafol absorbed (*q_t_* in mg/g) versus the contact time at six different initial concentrations. The amount of flutriafol adsorbed increased with contact time increasing. Most of the groups reached equilibrium after 90 min. As for diniconazole (Figure 6e), the same phenomenon was observed: the amount of diniconazole adsorbed also increased with absorption time increasing and reached equilibrium in 60 min.

Further investigation of the adsorption kinetics of these two compounds found they could be described by a pseudo-second-order model. The differential equation was as follows [42,43,44,45]:

*d_q_*/*d_t_* = *k* (*q_e_* – *q_t_*)^2^, (2)

1/(*q_e_* – *q_t_*) = 1/*q_e_* + *kt* (3)

*t*/*q_e_* = 1/(*k q_e_*^2^) + *t*/*q_e_* (4)

where *q_e_* was the amount of compound adsorbed at equilibrium (mg/g); *q_t_* was the amount of compound adsorbed at time *t* (mg/g); and *k* was the equilibrium rate constant of pseudo-second order sorption (g.mg^−1^.min^−1^). Integrating Equation (2) for the boundary conditions *t* = 0 to *t* = *t* and *q_t_* = 0 to *q_t_* = *q_t_* gives Equation (3), it could be rearranged to obtain a linear form of Equation (4).

Figure 6c,f showed the pseudo-second-order kinetics of flutriafol and diniconazole adsorption onto β-CD polymer, respectively. Values of *k* and *q_e_* were obtained from the intercept and slope of the plot of *t*/*q_t_* against t, listed in Table 2. It was obviously observed that the kinetics of flutriafol and diniconazole adsorption on β-CD polymer follows this model, with regression coefficients between 0.993 and 0.999. The calculated values agreed with the experimental data further indicated that the adsorption belonged to the second-order kinetics. The results showed the favorable adsorption performance of the materials for triazole pesticides, and the adsorption equilibrium could be reached quickly. Based on the results, flutriafol was selected as the model to evaluate the adsorption isotherm and the adsorption performance of P-MCB.

An adsorption isotherm represents the relationship existing between the amount of compound adsorbed and the compound concentration remaining in the solution. Adsorption equilibrium was established when the amount of compound adsorbed onto the material was equal to the amount desorbed. There were several isotherm equations available for analyzing experimental adsorption equilibrium data, including the Freundlich, Langmuir, Temkin, and so on [46,47]. There was a nonlinear relationship between the adsorption amount of flutriafol (*q_e_* (mg·L^−1^)) and the equilibrium concentration (*C_e_* (mg·L^−1^)) in the solution at different temperatures. In order to fit the experimental data, Freundich isotherm and Langmiur isotherm were involved. The regression coefficient (*R*^2^) of the Langmuir isothermal model ranged from 0.556 to 0.988, and that of the Freundlich model ranged from 0.993 to 0.999 in Table 2 and Table 3. This obviously indicated that the equilibrium experimental data was closer to the Friedrich isotherm model than the Langmuir isotherm model.

Freundlich model was used to describe the adsorption isotherm of flutriafol. The Freundlich isotherm was expressed by Equation (5), and a linear form of the Freundlich expression can be obtained by taking the logarithms of Equation (6).

*q_e_* = *K_F_*
*C_e_* ^1/*nF*^, (5)

ln *q_e_* = ln *K_F_* + (ln *C_e_*)/*n_F_* (6)

where *q_e_* was the amount of compound adsorbed at equilibrium (mg/g), *C_e_* was the equilibrium compound concentration in solution (mg/L), and *K_F_* was the Freundlich constant (mL/g), and 1/*n_F_* was the heterogeneity factor. *K_F_* and 1/*n_F_* were obtained from the plot of ln *q_e_* versus ln *C_e_* as the intercept value and the slope, listed in Table 4.

**Figure 7:**
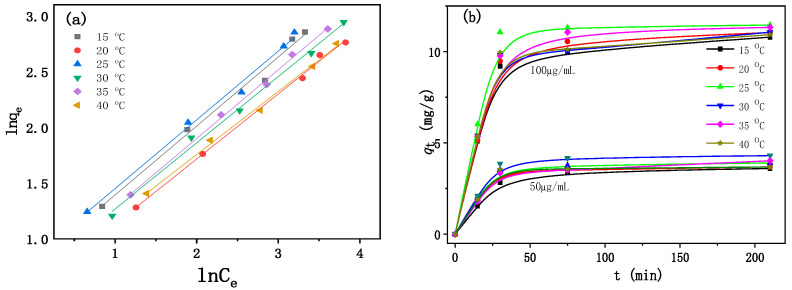
(**a**) Freundlich plots illustrating the linear dependences of ln*q_e_* on ln*C_e_* of flutriafol, (**b**) effect of temperature on the adsorption of flutriafol with different contact times.

Figure 7a shows the Freundlich plots of flutriafol with the initial concentration of 5 mg/L at different temperatures. It was obviously observed that the plots followed this model; five groups had regression coefficients higher than 0.99 except for the adsorption at 15 °C. The value of *n* was greater than 1.0, indicating favorable adsorption. Therefore, the adsorption isotherm of flutriafol on *β*-CD polymer could be expressed by the Freundlich equation.

Figure 7b shows the amount of flutriafol absorbed (*q_t_* in mg/g) versus the contact time at different temperatures. The amount of flutriafol adsorbed increased with contact time increasing. There was no significant influence of temperature on the adsorption efficiency at a low concentration of 50 μg/mL. The difference increased at a higher concentration of 100 μg/mL. Lower or higher temperatures were against adsorption. As for the sample of 100 μg/mL at 25 °C, it could reach equilibrium within 30 min.

**Figure 8:**
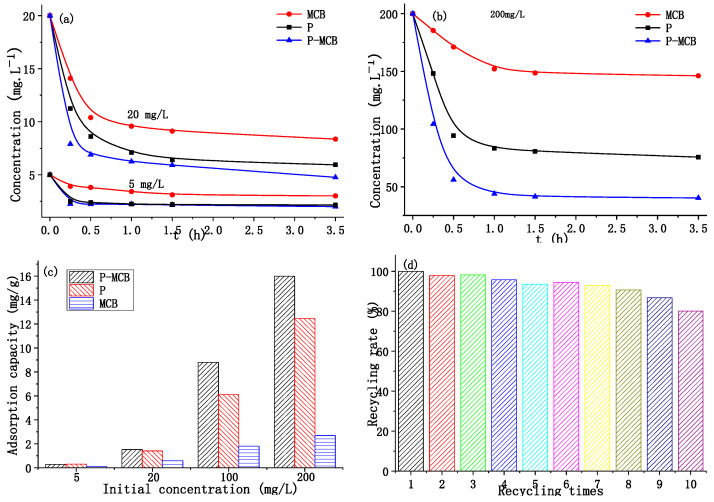
(**a**,**b**) Concentration of flutriafol in solution with contact time increasing for polymer (P), micro-ceramic balls (MCB), and polymer-loaded micro-ceramic balls (P-MCB) (conditions: flutriafol concentration = 200 mg/L, 100 mg/L, 20 mg/L, 5 mg/L; volume: 10 mL; Polymer mass = 100 mg, MCB = 2 g, P-MCB mass amounts to 100 mg polymer and 1.9 g MCB); (**c**) the adsorption of P-MCB, polymer, MCB in the flutriafol solution with an initial concentration of 200 mg/L, 100 mg/L, 20 mg/L, 5 mg/L; (**d**) the cyclic utilization ratio of P-MCB.

The adsorption capacity of the P-MCB, MCB, and polymer were evaluated, and the adsorption capacities of P-MCB, MCB and polymer were compared under different concentrations (5 mg/L, 20 mg/L, 200 mg/L) (Figure 8a,b). The adsorption of P-MCB, polymer, and MCB in the flutriafol solution with an initial concentration of 200 mg/L in 3.5 h was 15.98 mg/g, 12.44 mg/g, and 2.69 mg/g, respectively (Figure 8c). The adsorption capacity of P-MCB in 20 min was close to that of the Polymer in 1 h. The results indicated P-MCB group had a larger adsorption capacity and higher adsorption efficiency.

After adsorption, P-MCB filled in the column was washed with ethanol and dried under vacuum. Mass loss was less than 0.1%. P-MCB was reused, and their cyclic utilization ratio was evaluated (Figure 8d). A total of 10 cycles were tested, and there was no significant difference in the adsorption capacity between the first five circulations. The adsorption of P-MCB decreased after five times reusing; the adsorption decreased to 80.1% of initial capacity at the tenth cycling utilization. Of course, the recycling efficiency was also related to the volume of the adsorbed solution and the contact time of adsorption.

There were two reasons for the removal of these pesticides by P-MCB. Since the β-CD polymer contained numerous cyclodextrin, pollutants or various organic molecules could be captured through host-guest interactions, forming inclusion complexes. Moreover, the pesticides were removed due to the presence of nitrogen-containing groups, hydrophobic groups, and delocalized large π bonds on the benzene and pentagonal heterocycles of triazole pesticides. The hydrogen bonds, hydrophobic interactions, electrostatic interactions, and π-π stacking between P-MCB and these functional groups enabled P-MCB to remove triazole pesticides.

### 2.4. Thermodynamic Studies

In order to determine the physical and chemical properties of the entire adsorption process, the influence of temperature (thermodynamic study) on the adsorption of flutriafol and diniconazole by the adsorbent was monitored. By nonlinear fitting of adsorption isotherms (Freundlich isotherm models) at different temperatures, the thermodynamic parameters of the adsorption process at different temperatures, including Gibbs free energy, can be calculated (ΔG°/kJ mol^−1^), standard enthalpy change (ΔH°/kJ mol^−1^) and standard entropy change (ΔS°/J mol^−1^ K^−1^) [48]. The following was the thermodynamic parameter equation:

ΔG° = −RTln*Kc* (7)

ln*Kc* = −ΔH°/RT + ΔS°/R (8)

ΔG° = ΔH° − TΔS°(9)

where *Kc* was calculated by (*q_e_*/*C_e_*); *q_e_* was the amount of compound adsorbed at equilibrium (mg/g); *C_e_* was the equilibrium compound concentration in solution (mg/L); ΔS° and ΔH° can be calculated by the intercept and slope of ln (*q_e_*/*C_e_*) with the 1/T graph (Table 5). The negative value of ΔH° indicated that the adsorption of Flutriafol by β-CD polymer loaded micro-ceramic balls was exothermic. The negative value of ΔG° indicated the spontaneity of the adsorption process, as shown in Table 4. Furthermore, to a certain extent, the positive value of ΔS° corresponds to an irregular increase in randomness after the adsorption of Flutriafol.

### 2.5. Comparison of the Solid Phase Extraction Method of P-MCB with Other Methods

The solid phase extraction (SPE) method of P-MCB was compared with the other two represent methods, including magnetic solid phase extraction (MSPE) [2] and carbonized cellulose nanocrystal-based magnetic solid phase extraction (MSC-CNC-MSPE) [12], from the aspects of LOD, RSD, adsorption rate and extraction time. As shown in Table 6, the SPE of P-MCB had a lower LOD, lower RSD, and a relatively high adsorption capacity compared to these methods. In addition, the SPE method was simple to operate and did not require any special instruments.

## 3. Materials and Methods

### 3.1. Materials

β-Cyclodextrin (β-CD) (purity ≥ 99.0%) was provided by Aladdin Chemistry Co., Ltd. Citric acid and sodium dihydrogen phosphate were purchased from Sinopharm Chemical Reagent Co., Ltd. (Shanghai, China). Flutriafol and diniconazole were purchased from Chengdu Best Reagent Co., Ltd. (Chengdu, China). Micro-ceramic balls (MH-2, Diameter = 0.5 mm, aperture = 40~100 μm) were obtained from Pingxiang Huihua Packing Co., Ltd. (Pingxiang, China). All other reagents were of analytical grade without further treatment.

### 3.2. Measurements

FTIR spectra were recorded on a Bruker EQUINOX 55 spectrometer with the KBr technique. Powder X-ray diffraction measurements (XRD) were performed on a Bruker D8 Advance diffractometer using pressed pellets as samples with Cu Kα radiation (λ = 1.5418 Å) at a voltage of 40 kV and current of 200 mA. Scanning electronic microscopy (SEM) images were taken on a Nova NanoSEM 200 scanning electron microscope. HPLC was taken on an Agilent 1120 type liquid instrument using Inertsil ods-sp C18 chromatographic column. pH values were measured by Mattler Toledo pH meter (SG2-ELK).

### 3.3. Preparation of β-Cyclodextrin Polymer

#### 3.3.1. Preparation of β-Cyclodextrin-Citric Acid Conjugate

Citric acid (10.0 g) and sodium dihydrogen phosphate (3.39 g) were dissolved in deionized water (20 mL), followed by β-CD (10.0 g). The solution was stirred for 1 h at 103 °C firstly. Then, the solution was placed into an electric heating air-blowing driver for 0.5 h at 170 °C to obtain β-cyclodextrin polymer. The solid was washed with deionized water twice and dried to be a faint yellow transparent solid (yield: 9.7 g). It was ground into powder.

#### 3.3.2. Preparation of β-Cyclodextrin Polymer Loaded Micro-Ceramic Balls (P-MCB)

Citric acid (10.0 g) and sodium dihydrogen phosphate (3.39 g) were dissolved in deionized water (20 mL), followed by β-CD (10.0 g). The solution was stirred for 1 h at 103 °C. Then the solution was transferred to the culture dish, and 30 g micro-ceramic balls were added. The mixture was placed into a hot air cycle drying oven for 0.5 h at 170 °C. The β-cyclodextrin polymer loaded micro-ceramic balls were washed with deionized water to remove unreacted raw materials and dried.

### 3.4. Degradation of β-Cyclodextrin Polymer

50 mg β-CD polymer powder was added to a 10 mL solution with different pH values (pH = 2.24, 4.22, 6.70, 7.41, 8.54, 10.50) at 25 °C and 35 °C. pH value of these solutions was measured at prescribed time intervals until there was no distinguishable change, and the remaining solids were washed with deionized water and dried under vacuum to be weighed. The experiments were conducted in triplicate, and the results were demonstrated as mean ± SD.

### 3.5. Separation of Pesticides

Adsorption experiments of pesticides by β-CD polymer and P-MCB were performed in 20 mL flasks using 100 mg β-CD polymer. Different concentrations of flutriafol or diniconazole were added to the flasks. The samples were shaken at 110 rpm and at 15, 20, 25, 30, 35, 40 °C. At certain intervals, 0.1 mL suspension was sampled and filtered through a 0.45 mm Millipore glass fiber membrane. The pesticides in the filtrate were measured by HPLC. HPLC method for flutriafol is as follows: the mobile phase was 60% aqueous methanol, and the flow rate was 1.0 mL/ min. The detection wavelength was set at 220 nm, and the column was heated at 27 °C. HPLC method for diniconazole is as follows, the mobile phase was 80% aqueous methanol, and the flow rate was 1.0  mL/ min. The detection wavelength was set at 254 nm, and the column was heated at 40 °C.

Adsorption experiments of pesticides by P-MCB were performed as follows, 60 g P-MCB was filled in a glass column with a diameter of 20 mm, as shown in Figure 1. Different concentrations of pesticides were added to the column and held in the column for different times. At certain intervals, 0.1 mL solution was sampled and filtered through a 0.45 mm Millipore glass fiber membrane. The pesticides in the filtrate were measured by HPLC.

## 4. Conclusions

In this paper, β-CD polymer was synthesized by cross-linking of citric acid and loaded onto micro-ceramic balls to obtain the sorbents for the sorption of triazole pesticides. Both the β-CD polymer and β-CD polymer loaded micro-ceramic balls (P-MCB) were more suitable for neutral medium at low temperature, and less degradation of 0.2% ± 0.08 in 10 d for P-MCB in medium guaranteed the usage of P-MCB for solid-phase extraction. The influence of several parameters (kinetics, sorbent amount, pesticide concentration, temperature) on the adsorption capacity has been evaluated. The equilibrium data have been analyzed using the Freundlich equation. The kinetics of flutriafol and diniconazole adsorption on β-CD polymer follows the pseudo-second-order kinetics, with regression coefficients between 0.993 and 0.999. The results showed the favorable adsorption performance of the materials for triazole pesticides, and the adsorption equilibrium could be reached quickly. In addition, P-MCB had a large adsorption capacity of 15.98 mg/g in the flutriafol solution with an initial concentration of 200 mg/L in 3.5 h. The P-MCB also demonstrated satisfied recycling usage; 80.1% of initial capacity was obtained even at the tenth cycling utilization.

## Figures and Tables

**Table 1:** Degradation efficiency (DE%) of β-CD polymer at different media for 10 d.

Adsorbents	T (°C)	pH Value of Initial Solution				
		4.2	7.0	7.7	8.5	10.5
β-CD polymer	25	10.4 ± 0.14%	0.9 ± 0.12%	3.3 ± 0.20%	12.1 ± 0.11%	12.8 ± 0.23%
	35	23.2 ± 0.11%	6.8 ± 0.21%	11.6 ± 0.15%	22.0 ± 0.14%	22.9 ± 0.20%
P-MCB	25	3.2 ± 0.13%	0.2 ± 0.08%	0.6 ± 0.21%	2.9 ± 0.12%	3.1 ± 0.17%
	35	7.7 ± 0.18%	1.7 ± 0.11%	3.9 ± 0.19%	6.2 ± 0.31%	7.2 ± 0.25%

W1, initial weight; W2, remained weight; DE%, degradation efficiency; DE% = (W1 – W2)/W1 × % P-MCB, β-CD polymer loaded micro-ceramic balls.

**Table 2:** Freundlich adsorption kinetic parameters of flutriafol and diniconazole onto the adsorbents at 25 °C. (a, experimental values; b, calculated values).

Compounds	Initial *C_i_* (mg/L)	Parameters and *R*^2^ Values at Different Concentrations			
		*q_e_ ^a^*, mg/g	*k*_2_, g/(mg.min)	*R* ^2^	*q_e_ ^b^*, mg/g
Flutriafol	5	0.382	0.171	0.994	0.359
	20	1.897	0.031	0.993	1.760
	50	4.009	0.044	0.999	3.903
	100	8.048	0.034	0.999	7.888
	150	11.669	0.025	0.999	11.459
	200	16.204	0.012	0.999	15.962
Diniconazole	1	0.098	−24.475	0.999	0.098
	5	0.937	41.678	0.999	0.937
	20	1.762	0.562	0.999	1.757
	50	4.556	0.071	0.999	4.469

**Table 3:** Langmuir adsorption kinetic parameters of flutriafol and diniconazole onto the adsorbents at 25 °C.

Compounds	Initial *C_i_* (mg/L)	Parameters and *R*^2^ Values at Different Concentrations		
		*1*/*q_e_*, mg/g	*R* ^2^	1/*C_e_*, L/mg
Flutriafol	5	0.0089	0.988	0.2425
	20	0.0441	0.927	0.0747
	50	0.1416	0.864	0.0348
	100	0.3107	0.685	0.0187
	150	0.4653	0.702	0.0125
	200	0.7143	0.924	0.0108
Diniconazole	1	0.0033	0.556	25
	5	0.0312	0.824	1.5625
	20	0.0546	0.828	0.2169
	50	0.1167	0.763	0.0715

**Table 4:** Adsorption isotherm parameters for flutriafol onto the adsorbents at different temperatures.

Freundlich Isotherm							
Compounds	Parameters	Adsorption at Different Temperature					
		15 °C	20 °C	25 °C	30 °C	35 °C	40 °C
Flutriafol	*n*	1.616	1.713	1.622	1.683	1.623	1.767
	*K_F_*	0.775	0.549	0.835	0.674	0.668	0.625
	*R* ^2^	0.9898	0.9975	0.9926	0.9943	0.9971	0.9965

**Table 5:** Thermodynamic parameters at different temperatures.

Compounds	Parameters	Different Temperatures					
		15 °C	20 °C	25 °C	30 °C	35 °C	40 °C
Flutriafol	ΔG° (kJ mol^−1^)	−1.081	−0.060	−1.452	−0.611	−0.525	−0.051
	ΔH° (kJ mol^−1^)	−0.489					
	ΔS° (/J mol^−1^ K^−1^)	0.460					

**Table 6:** Comparison of the SPE method of P-MCB with other methods.

Methods	Instrumental Technique	Sample	LOD (μg/L)	RSD (%)	Adsorption Capacity (%)	Ref.
SFE of P-MCB	HPLC	Water	0.0004–0.0005	0.08–0.31	80.1–100	This work
MSPE	HPLC-MS/MS	Water	0.05–0.1	1.5–9.1	72.3–91.53	[2]
MPC-CNC-MSPE	UHPLC-MS/MS	Water	0.003–0.007	2.0–15.7	73.7–117.1	[12]

SPE: solid phase extraction; MSPE: magnetic solid phase extraction; MSC-CNC-MSPEM: carbonized cellulose nanocrystal-based magnetic solid phase extraction.

## Data Availability

Data are contained within the article.
